# Influence of Poly(L-Lactic Acid) Nanofibers and BMP-2–Containing Poly(L-Lactic Acid) Nanofibers on Growth and Osteogenic Differentiation of Human Mesenchymal Stem Cells

**DOI:** 10.1100/tsw.2008.163

**Published:** 2008-12-25

**Authors:** Markus D. Schofer, Susanne Fuchs-Winkelmann, Christian Gräbedünkel, Christina Wack, Roland Dersch, Markus Rudisile, Joachim H. Wendorff, Andreas Greiner, Jürgen R. J. Paletta, Ulrich Boudriot

**Affiliations:** ^1^ Department of Orthopedics, University Hospital of Marburg, D-35043 Marburg, Baldingerstraße, Germany; ^2^ Department of Chemistry, University of Marburg, D-35032 Marburg, Hans-Meerwein-Straße, Germany; ^3^ Department of Orthopedics, Sankt-Elisabeth-Hospital Gütersloh, D-33332 Gütersloh, Kattenstroth 103, Germany

**Keywords:** nanofibers, tissue engineering, human mesenchymal stem cells, PLLA, BMP

## Abstract

The aim of this study was to characterize synthetic poly-(L-lactic acid) (PLLA) nanofibers concerning their ability to promote growth and osteogenic differentiation of stem cells in vitro, as well as to test their suitability as a carrier system for growth factors. Fiber matrices composed of PLLA or BMP-2–incorporated PLLA were seeded with human mesenchymal stem cells and cultivated over a period of 22 days under growth and osteoinductive conditions, and analyzed during the course of culture, with respect to gene expression of alkaline phosphatase (ALP), osteocalcin (OC), and collagen I (COL-I). Furthermore, COL-I and OC deposition, as well as cell densities and proliferation, were analyzed using fluorescence microscopy. Although the presence of nanofibers diminished the dexamethasone-induced proliferation, there were no differences in cell densities or deposition of either COL-I or OC after 22 days of culture. The gene expression of ALP, OC, and COL-I decreased in the initial phase of cell cultivation on PLLA nanofibers as compared to cover slip control, but normalized during the course of cultivation. The initial down-regulation was not observed when BMP-2 was directly incorporated into PLLA nanofibers by electrospinning, indicating that growth factors like BMP-2 might survive the spinning process in a bioactive form.

### INTRODUCTION

The reconstruction of large bony defects after injury or tumor resection often requires the use of graft material. The use of autologous bone grafts has been regarded as the gold standard of bone repair and regeneration. Although there are many advantages, there are also major drawbacks, such as donor site morbidity and availability[1]. These complications affect approximately 10% of patients[2]. To overcome these drawbacks, artificial scaffolds based on synthetic biomaterials, such as metals, ceramics, polymers, and composites, have been developed[1,3]. In order to engineer the ideal bone graft material, substances that are capable of triggering osteogenesis must be included[4]. Therefore, a three-dimensional (3-D) scaffold should function as a carrier for either growth factors or cells[5,6]. Here, scaffolds based on nanofibers, produced by electrospinning, offer great advantages[7,8]. These fibers mimic the extracellular matrix[9,10] and allow the differentiation of human mesenchymal stem cells (hMSC) towards osteoblasts.

These nanofibers can be produced by a broad spectrum of polymers including biocompatible as well as biodegradable polymers, such as poly(glycolic acid) (PGA), poly(L-lactic acid) (PLLA), poly(ε caprolactone) (PCL), polyurethanes, polyphosphazenes, collagen, gelatin, and chitosan, as well as copolymers from the corresponding monomers in various compositions[10,11]. This allows the production of a broad spectrum of nanofiber-based scaffolds with different mechanical and biophysical properties. It also offers the chance to incorporate growth factors in order to use the nanofibers as a drug carrier system. Here, the cytokines of the BMP family seem to offer a chance to enhance bone regeneration by promoting a high level of osteoblast activity[12]. These growth factors can be incorporated into polylactic polymers without losing their bioactivity using organic solvents[13,14,15,16]. This might implicate that BMPs have a good chance of surviving the electrospinning process. Therefore, the aim of this study was to characterize the osteogenic differentiation of hMSC on PLLA nanofibers and to analyze whether BMP-2 can be incorporated in a bioactive form into these PLLA nanofiber constructs.

### MATERIAL AND METHODS

#### Construction of Nanofibers and Characterization

The preparation of PLLA nanofibers by electrospinning has been reported in detail earlier[17]. Briefly, a 4% (w/w) PLLA (Resomer L210, Boeringer Ingelheim Germany) solution in dichloromethane was prepared at room temperature by stirring overnight until a homogenous solution was obtained. Samples of nonwoven PLLA nanofibers were fixed on 19-mm cover slips for cell culture experiments.

In order to incorporate BMP-2 into the nanofibers, 25 µg of lyophilized BMP-2 (Reliatech, Braunschweig Germany) was dissolved in 125 µl of 50 m*M* acetic acid and stabilized by the addition of 25 µl of fetal calf serum (FCS). This mixture was emulsified in 2.5 ml of a 4% PLLA-dichloromethane solution over a period of 1 min, using a vortex mixer (MS 2 Minishaker IKA®; 2500 rpm). This solution was sufficient for the spinning of 50 cover slips with an approximate amount of 0.2–0.5µg BMP-2, depending on the spinning and fiber collection process.

The spinning process was performed at a flow rate of 14 µl/min with an applied voltage of 20–30 kV and a distance of 15 cm. In some experiments, BMP was replaced by a FITC-labeled protein in order to analyze the distribution within the nanofibers.

#### Contact Angles

Static contact angles of water were measured using the sessile drop method with a G10 Drop Shape Analysis System (Krüss, Hamburg, Germany) and calculated using Data Physics SCA20 Contact Angle Analyzer Software.

### Scanning Electron Microscopy

For scanning electron microscopy (SEM), samples were sputter coated with gold in an AUTO-306 (BOC Edwards, Crawley, Sussex, U.K.) high-vacuum coating system and examined in a SE microscope (S-4100, Hitachi Ltd., Tokyo, Japan) at an accelerating voltage of 5 kV in the SE mode.

### hMSC Isolation, Characterization, and Culture hMSC were obtained from consenting patients with the approval of the institutional review board. The indication for surgery was primary osteoarthritis of the hip with increasing pain, decreased range of motion, and signs of progressive osteoarthritis in radiographs. The patients had no evidence of other bone or autoimmune diseases. The routinely removed bone was obtained from the proximal femur while preparing the implant bed. hMSC were isolated and cultured according to the preparation of Pittenger et al.[18], with minor modification as described by Brendel et al.[19].

Within the experiments, hMSC preparations negative for CD34 and CD40 and positive for the stem cell markers CD90 and CD105 were used. In order to ensure the osteoinductive potential of the obtained cells, gene expression of alkaline phosphatase, in response to dexamethasone, was determined prior to the experiments.

For experiments, hMSC were seeded at a density of $3 \times 10^4$ cells/cm² on cover slips or cover slips coated with either PLLA or BMP-2–containing nanofibers in growth medium (DMEM), with low glucose and glutamine (PAA, Linz, Austria), supplemented with 10% FCS from selected lots (Stem Cell Technologies, Vancouver, Canada) and 1% penicillin/streptomycin. In some experiments, osteogenic differentiation was induced according to Jaiswal et al. after an initial proliferation phase of 3 days[20]. The medium was replaced every 2$^{nd}$ day of culture.

### Gene Expression Analysis

RNA was extracted from cell layers at days 4, 10, and 22 (after introduction of osteoinductive conditions) using RNeasy Mini Kit (Qiagen GmbH, Hilden, Germany) according to the manufacturer and quantified spectrometrically. Starting from 1 µg of RNA, 20 µl of cDNA were synthesized using Omniscript reverse transcriptase and oligo-dT primer in the presence of dNTP (Qiagen GmbH, Hilden, Germany). Quantitative RT-PCR reactions were performed and monitored using a Mastercycler® ep realplex Detection System (Eppendorf, Hamburg, Germany) and RealMaster Mix CyberGreen (Eppendorf, Hamburg, Germany). Genes of interest were analyzed in cDNA samples (5 µl for a total volume of 50 µl/reaction) using the DeltaDeltaCt method and CyberGreen. Primers (purchased at TIB Biomol, Berlin, Germany), cycle temperatures, and incubation times for human alkaline phosphatase (ALP), osteopontin (OP), collagen I (COL-I), osteocalcin (OC), and 18s rRNA were previously described[21,22,23]. Purity of the single PCR products was verified by melting point analysis.

### Immunofluorescence Microscopy

Samples obtained at day 22 were fixed in acetone/methanol, washed with PBS (3×), and exposed to blocking buffer (1% donkey serum albumin PBS) for a further 30 min at room temperature in order to minimize nonspecific absorption of the antibodies. After another wash in PBS (3×), the cells were incubated with primary antibodies against COL-I (Abcam, Ab6308, Cambridge, U.K.), OC (Acris, BP710, Hiddenhausen, Germany), OP (Abcam, Ab8448, Cambridge, U.K.), and Ki-67 (Darko, Hamburg, Germany).

Visualization was done, after washing in PBS (3×) using cy-2– or cy-3–conjugated secondary antibody (Dianova, Hamburg, Germany) at room temperature (1 h). The slices were stained with DAPI and embedded in mounting medium. Fluorescence microscopy was done using a Leica DM5000. Microphotographs of at least three different areas were made at a primary magnification of 20-fold high-power field (HPF). Intensity of fluorescence was determined using Quips analysis software. Total cell count of DAPI-stained nuclei was obtained. The proliferation index was calculated on a ratio of Ki-67 positive vs. total cells. Patient series were analyzed by independent observers.

### Statistics

All values were expressed as mean ± standard error of different patients as indicated in the text and compared using students' t-test or ANOVA with Bonferroni as a posthoc test. Values of $p < 0.05$ were considered to be significant. Significant values were marked with *, whereas $^{+}$ indicates tendency within the patient series.

## RESULTS

### Characterization of PLLA and BMP-2–Incorporated PLLA Nanofibers

SEM of electrospun PLLA nanofibers revealed a 3-D nonwoven network with a diameter of 775 ± 294 nm. Fibers were porous in structure (Fig. 1A) and had a contact angle of 124.6 ± 5.7°. In aqueous solutions, the PLLA fibers were stable over a period of 30 days.

In contrast, the incorporation of BMP-2 resulted in a reduced pore development on nanofiber surface and the formation of spindle-shaped beads in more-or-less regular intervals (Fig. 1B).

Furthermore, BMP-2–containing nanofibers showed a decrease in the overall diameter of the fibers (600 ± 343 nm). The contact angle was reduced to 115.2 ± 5.5° by the incorporation of BMP-2 compared to PLLA nanofibers.

When FITC-labeled BSA was used as a model for the incorporation of proteins by electrospinning, fluorescence microscopy showed evidence that – in case of FITC-labeled BSA – fluorescence accumulated within the slubs mentioned above (Figs. 1C, D).

### Growth and Proliferation of hMSC Cultured on Nanofibers

In order to describe the biological effects, we first analyzed the effect of PLLA and PLLA-BMP nanofibers on proliferation of hMSC, and compared it to that of hMSC cultured on cover slips. As shown in Fig. 2B, the addition of dexamethasone resulted in an increase of the proliferation rate of stem cells cultured on cover slips. This is altered by the presence of PLLA as well as PLLA-BMP nanofibers. Here the proliferation rate, determined by Ki67 staining, decreased at day 10 ($p = 0.002$; n = 3) and day 22 ($p = 0.000$; n = 3). Nevertheless, this effect on PLLA nanofibers was not reflected in cell densities. Under osteoinductive conditions, there was a significant time-dependent increase in cell density within 22 days, regardless of whether cells were cultured on glass ($p = 0.000$; n = 7), PLLA ($p = 0.000$; n = 7), or PLLA-BMP ($p = 0.001$; n = 4) nanofibers (Fig. 2A). Furthermore, there were no significant differences between cell densities on glass and on either PLLA or PLLA-BMP at any point of time.

When cultured under growth conditions (Fig. 2C), cell densities only increased until day 10 when cultured on cover slips ($p = 0.013$; n = 7) or PLLA nanofibers ($p = 0.007$; n = 7), and remained constant until the end of culture. On PLLA-BMP nanofibers, no significant increase in cell densities was observed during the 22 days of culture. Furthermore, there were no differences in proliferation (Fig. 2 D) between the groups under growth conditions.

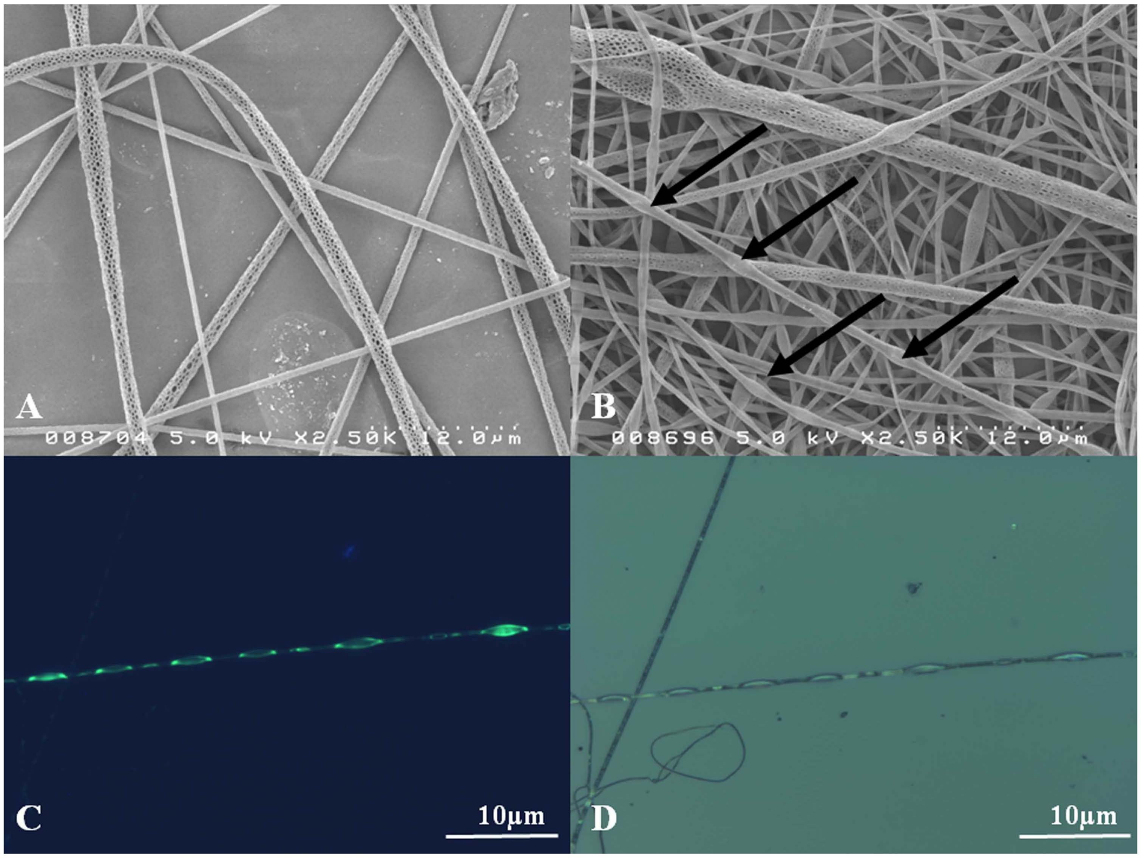

**FIGURE 1.** Fiber characterization. SEM analysis of PLLA (A) and BMP-2–enwoven PLLA nanofibers (B). Arrows indicate the formation of spindle-shaped beads when BMP-2 is enwoven. Fluorescent microscopy (C) and phase contrast microscopy (D) indicates the accumulation of FITC-labeled protein within these slubs. Scale bar represents 10μm.

### Influence of PLLA Nanofibers on the Differentiation and Matrix Formation of hMSC

In order to examine the influence of PLLA nanofibers on hMSC differentiation towards osteoblasts, we compared the expression levels of cells grown on cover slips with cells grown on PLLA nanofibers using the DeltaDeltaCt method. Single extreme values of the series, influencing the mean but not the median, were omitted from the calculation. Regardless of whether the cells were cultured under growth (n = 7) or osteoinductive (n = 5) conditions, the presence of PLLA nanofibers resulted in an initial down-regulation of genes associated with osteoblast lineage. When cultured under growth conditions (Fig. 3A), ALP (0.52-fold; $p = 0.000$), COL-I (0.38-fold; $p = 0.000$), and OC (0.72-fold; $p = 0.022$) gene expressions were reduced. Under osteoinductive conditions, this finding was true for ALP (0.54-fold $p = 0.002$), COL-I (0.63-fold; $p = 0.008$), and for OC in the majority (four of five) of patient series (Fig. 3B). However, the gene expression of the osteoblast lineage normalized or slightly increased on PLLA nanofibers independent of the culture conditions during the course of cultivation.

These results were consistent with those obtained by immune staining for COL-I and OC (Figs. 3C, D) as well as OP (Figs. 2E, F). Although there was a broad interpatient variability in the immune staining, we found less COL-I and OC stained areas after 10 days of culture on PLLA nanofibers (Fig. 3D) and a slight increase after 22 days (Fig. 3F) compared to cover slips control.

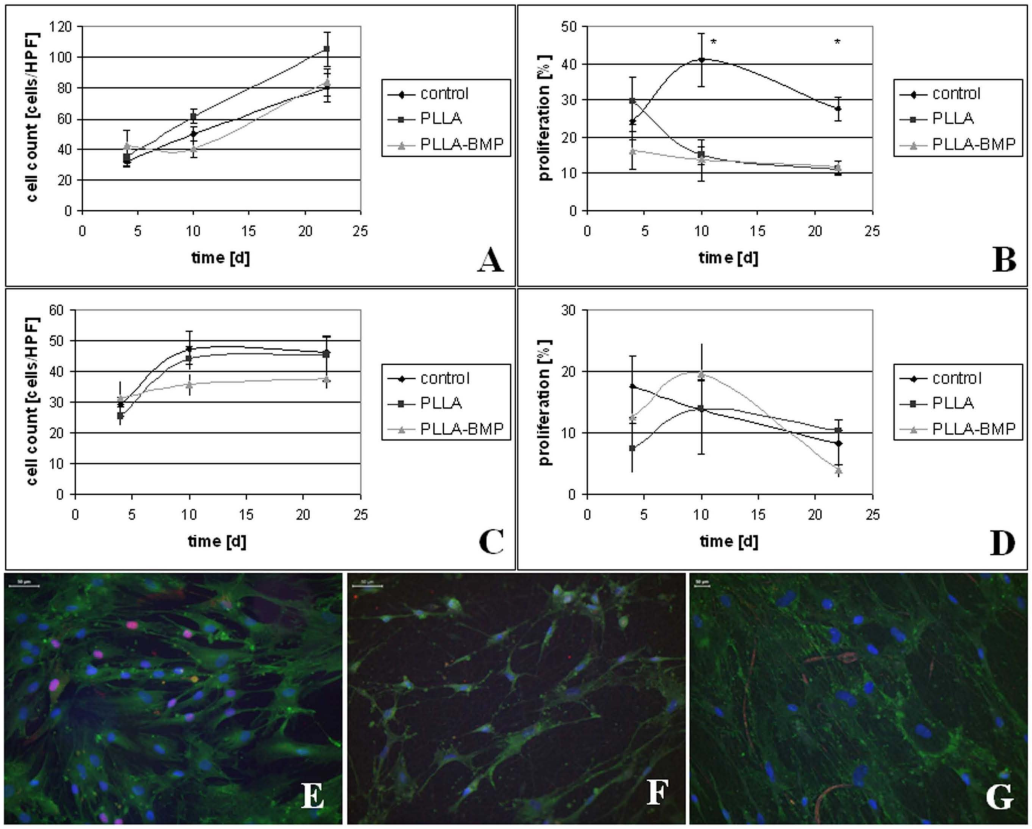

**FIGURE 2.** Influence of PLLA and PLLA-BMP nanofibers on growth and proliferation of hMSC cultured under growth and osteoinductive conditions. Time course of cell densities cultured under osteoinductive (A) and growth conditions (C). Time course of proliferation obtained under osteoinductive (B) and growth conditions (D). Immunofluorescence microphotographs of proliferation (red) and OP deposition (green) of hMSC cultured under osteoinductive conditions (day 10) on cover slips (E), PLLA nanofibers (F), and BMP-2–enwoven PLLA nanofibers (G). Scale bar represents 50 µm.

### Bioactivity of BMP-2–Enwoven Nanofibers

In order to overcome the PLLA-induced down-regulation of genes involved in differentiation, BMP-2 was incorporated into the PLLA nanofibers by electrospinning ($n = 4$). When stem cells were cultured under growth conditions on BMP-2–enwoven PLLA nanofibers, the initial gene expression of ALP (4.6-fold; $p = 0.002$), OC (5.4-fold; $p = 0.002$), and COL-I (2.7-fold; $p = 0.002$) was significantly increased compared to the gene expression of cells cultured on PLLA fibers alone (Fig. 4A). As shown in Fig. 4B, the presence of dexamethasone (osteoinductive conditions) potentiates the BMP-2 effect, resulting in an increase of ALP (11.6–fold; $p = 0.003$), COL-I (10.4-fold; $p = 0.001$), and OC (12.1-fold; $p = 0.006$). In the course of experiment, this effect diminished irrespective of culture conditions.

### DISCUSSION

PLLA is a biocompatible, biodegradable, and FDA-approved polymer commonly used as pins, screws, or membranes in bone reconstructive surgery[24,25,26,27]. As reported earlier, PLLA can easily be electrospun to a 3-D nonwoven network[28,29]. These constructs are appropriate as a matrix for osteoblast growth as well as for the osteogenic differentiation of hMSC in principle[17,30,31]. Nevertheless, little is known about the characteristics of hMSC differentiation on PLLA nanofibers over time.

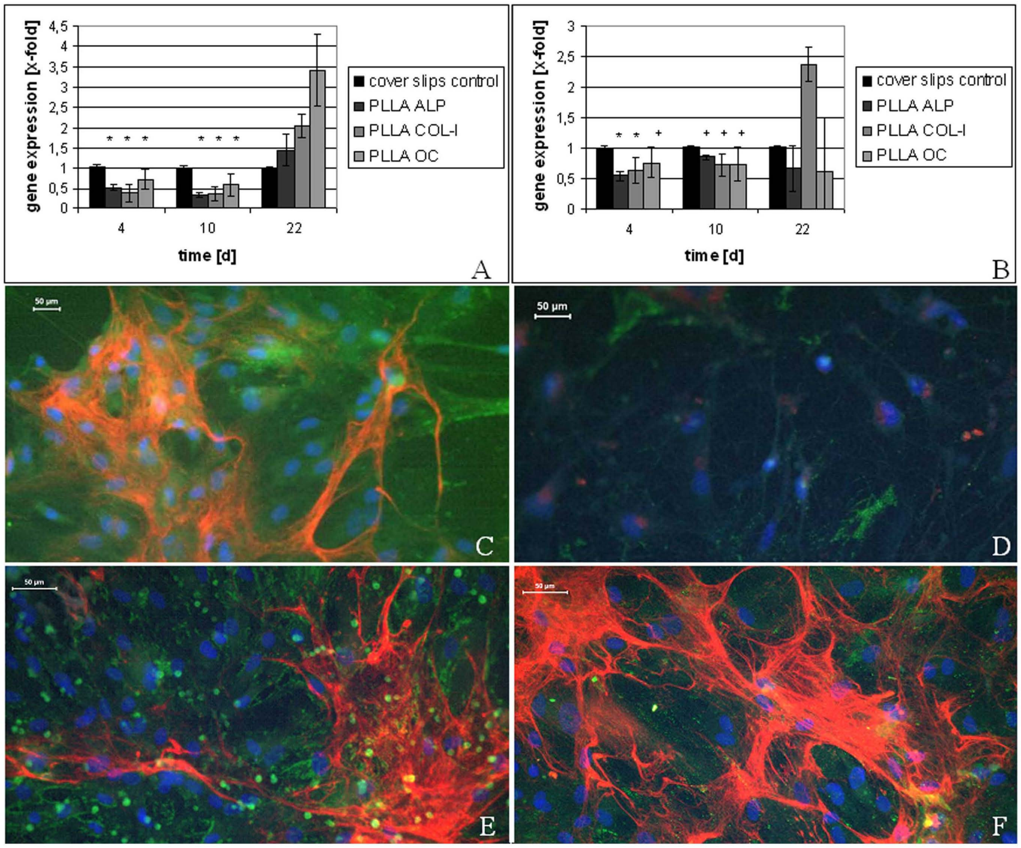

**FIGURE 3.** Influence of PLLA on gene expression and matrix deposition of hMSC cultured under growth and osteoinductive conditions. Time course of gene expression of hMSC cultured under growth (A) and osteoinductive conditions (B) on PLLA nanofibers compared to cover slips control. Immunofluorescence microphotographs of OC (green) and COL-I (red) deposition after differentiation of hMSCs on cover slips over a period of 10 (C) and 22 (E) days on PLLA nanofibers (D 10 days; F 22 days), respectively. Scale bar represents 50 µm. (ALP, COL-I, and OC gene expression of the cover slips control is set to 1 and therefore represented by only one bar.)

In this study, we first analyzed the growth and differentiation of hMSC on PLLA nanofibers in detail. While the addition of dexamethasone resulted in an increase of proliferation, the presence of PLLA nanofibers reduced the proliferation rates during the course of cultivation. In the case of osteoblast proliferation, the inhibitory effect on PLLA polymer[32,33] as well as PLLA nanofibers produced by phase separation[34] has been described. However, this reduced proliferation had no impact on the cell densities over a period of 22 days. This finding was independent of the culture conditions. We interpret this finding to mean that the 3-D PLLA nanofiber matrix provides a more efficient surface for cell propagation compared to the 2-D glass surface and could therefore be suitable for tissue engineering.

On the other hand, the presence of PLLA nanofibers influenced the expression of genes associated with the osteoblast lineage. Although there was a time-dependent up-regulation in gene expression independent of the surface (data not shown) in response to dexamethasone, gene expression was altered by the presence of unmodified PLLA nanofibers. Regardless of the culture conditions (growth or osteoinductive), gene expression of ALP, OC, and COL-I was reduced during the early stages of culture compared to cells cultured on cover slips. Although other methods were used, similar data were presented for PLLA nanofibers[35] as well as nanofibers made of other polymers[36,37], indicating that this phenomenon is influenced by structure more than by chemical composition. Nevertheless, gene expression normalized over the period of cultivation. Here immunofluorescence analysis of COL-I and OC

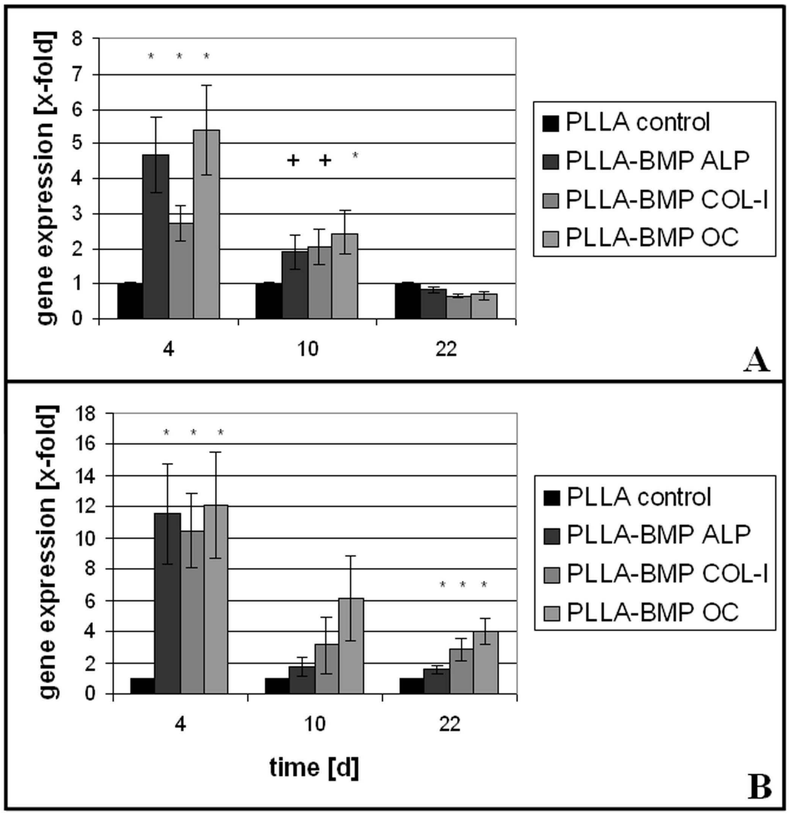

**FIGURE 4.** Influence of BMP-enwoven PLLA nanofibers on gene expression of osteoblast markers. Time course of gene expression of hMSC cultured under growth (A) and osteoinductive conditions (B) on BMP–enwoven PLLA nanofibers compared to PLLA nanofibers. (ALP, COL-I, and OC gene expression of the PLLA control is set to 1 and therefore represented by only one bar.)

resulted in analogous staining intensities compared to cover slips. Therefore, the PLLA nanofiber constructs seem to be suitable matrices for the filling of bone defects.

In order to overcome the initial down-regulation of osteoblast marker genes, and to provide the PLLA nanofibers with osteoinductive properties, human recombinant BMP-2 was incorporated.

BMP-2 is known to promote osteoblast activity[12] and can be incorporated into PLLA polymers using organic solvents without losing its bioactivity[13,14,15,16]. In our study, we used PLLA nanofibers due to their stability when incubated in physiological solutions over time[38]. Although PLLA is known to possess poor protein-release properties[29,39], during the electrospinning process, hydrophilic compounds were accumulated on the fiber surface[40]. The large surface and the porous structure of PLLA nanofibers might facilitate contact to the BMP receptor of the growing cell. This takes for granted that the incorporation of the growth factor has no impact on the PLLA fiber.

Although the porous surface was diminished, we found similar diameter and contact angles of PLLA nanofibers after incorporation of BMP-2. Both can be explained by the occurrence of beads in the case of growth factor–loaded fibers. These spindle-shaped beads were thought to carry BMP-2, indicating that distribution is not equable within the fibers. Such nonuniform distribution has been described earlier[41].

When hMSC were cultured on BMP-enwoven nanofibers, we found an increase in the expression of genes associated with osteoblast lineage compared to pure PLLA nanofibers. Moreover, this effect was more obvious in the presence of dexamethasone. Due to the fact that BMP-2 is known to enhance the dexamethasone-induced osteogenic differentiation of hMSC[12], we interpret this to mean that BMP-2 is present bioactively on the PLLA nanofiber surface and is recognized by stem cells. Here the use of hydroxylapatite, which ensures the structural integrity[42] and bioactivity[43] of BMP-2 when incorporated into Polylactic-co-Glycolid (PLGA) scaffolds, seems to be not necessary.

Therefore besides the coupling of growth factors on the surface of nanofibers[44,45] or incorporating into self-assembled peptide-amphiphile nanofibers[46], the incorporation via electrospinning can be a suitable tool in bone tissue engineering.

### ACKNOWLEDGMENTS

This work was supported by the Deutsche Forschungsgemeinschaft (German Research Foundation, Grant No. BO 3065/1-1).

## References

1. Kneser, U., Schaefer, D.J., Polykandriotis, E., and Horch, R.E. (2006) Tissue engineering of bone: the reconstructive surgeon's point of view. *J. Cell. Mol. Med.* **10,** 7–19.
2. Arrington, E.D., Smith, W.J., Chambers, H.G., Bucknell, A.L., and Davino, N.A. (1996) Complications of iliac crest bone graft harvesting. *Clin. Orthop. Relat. Res.* **(329),** 300–309.
3. Khan, Y., Yaszemski, M.J., Mikos, A.G., and Laurencin, C.T. (2008) Tissue engineering of bone: material and matrix considerations. *J. Bone Joint Surg. Am.* **90(Suppl 1),** 36–42.
4. Meijer, G.J., de Bruijn, J.D., Koole, R., and van Blitterswijk, C.A. (2007) Cell-based bone tissue engineering. *PLoS Med.* **4,** e9.
5. Damien, C.J. and Parsons, J.R. (1991) Bone graft and bone graft substitutes: a review of current technology and applications. *J. Appl. Biomater.* **2,** 187–208.
6. Lane, J.M., Tomin, E., and Bostrom, M.P. (1999) Biosynthetic bone grafting. *Clin. Orthop. Relat. Res.* **(367 Suppl),** S107–117.
7. Ashammakhi, N., Ndreu, A., Yang, Y., Ylikauppila, H., and Nikkola, L. (2008) Nanofiber-based scaffolds for tissue engineering. *Eur. J. Plast. Surg.,* online first.
8. Zhang, Y., Lim, C.T., Ramakrishna, S., and Huang, Z.M. (2005) Recent development of polymer nanofibers for biomedical and biotechnological applications. *J. Mater. Sci. Mater. Med.* **16,** 933–946.
9. Li, W.J., Laurencin, C.T., Caterson, E.J., Tuan, R.S., and Ko, F.K. (2002) Electrospun nanofibrous structure: a novel scaffold for tissue engineering. *J. Biomed. Mater. Res.* **60,** 613–621.
10. Liao, S., Li, B., Ma, Z., Wei, H., Chan, C., and Ramakrishna, S. (2006) Biomimetic electrospun nanofibers for tissue regeneration. *J. Biomed. Mater.* **1,** R45–53.
11. Greiner, A. and Wendorff, J.H. (2007) Electrospinning: a fascinating method for the preparation of ultrathin fibres. *Angew. Chem. Int. Ed.* **46,** 5670–5703.
12. Jäger, M., Fischer, J., Dohrn, W., Li, X., Ayers, D.C., Czibere, A., Prall, W.C., Lensing-Höhn, S., and Krauspe, R. (2008) Dexamethasone modulates BMP-2 effects on mesenchymal stem cells in vitro. *J. Orthop. Res.* 26(11), 1440–1448.
13. Schmidmaier, G., Wildemann, B., Cromme, F., Kandziora, F., Haas, N.P., and Raschke, M. (2002) Bone morphogenetic protein-2 coating of titanium implants increases biomechanical strength and accelerates bone remodeling in fracture treatment: a biomechanical and histological study in rats. *Bone* **30,** 816–822.
14. Schmidmaier, G., Lucke, M., Schwabe, P., Raschke, M., Haas, N.P., and Wildemann, B. (2006) Collective review: bioactive implants coated with poly(D,L-lactide) and growth factors IGF-I, TGF-beta1, or BMP-2 for stimulation of fracture healing. *J. Long Term Eff. Med. Implants* **16,** 61–69.
15. Raschke, M., Wildemann, B., Inden, P., Bail, H., Flyvbjerg, A., Hoffmann, J., Haas, N.P., and Schmidmaier, G. (2002) Insulin-like growth factor-1 and transforming growth factor-beta1 accelerates osteotomy healing using polylactide-coated implants as a delivery system: a biomechanical and histological study in minipigs. *Bone* **30,** 144–151.
16. Schmidmaier, G., Wildemann, B., Lubberstedt, M., Haas, N.P., and Raschke, M. (2003) IGF-I and TGF-beta 1 incorporated in a poly(D,L-lactide) implant coating stimulates osteoblast differentiation and collagen-1 production but reduces osteoblast proliferation in cell culture. *J. Biomed. Mater. Res. B Appl. Biomater.* **65,** 157–162.
17. Boudriot, U., Dersch, R., Goetz, B., Griss, P., Greiner, A., and Wendorff, J.H. (2004) Elektrogesponnene Poly-L-Laktid-Nanofasern als resorbierbare Matrix fur Tissue-Engineering. *Biomed. Tech. (Berl.)* **49,** 242–247.
18. Pittenger, M.F., Mackay, A.M., Beck, S.C., Jaiswal, R.K., Douglas, R., Mosca, J.D., Moorman, M.A., Simonetti, D.W., Craig, S., and Marshak, D.R. (1999) Multilineage potential of adult human mesenchymal stem cells. *Science* **284,** 143–147.

19. Brendel, C., Kuklick, L., Hartmann, O., Kim, T.D., Boudriot, U., Schwell, D., and Neubauer, A. (2005) Distinct gene expression profile of human mesenchymal stem cells in comparison to skin fibroblasts employing cDNA microarray analysis of 9600 genes. *Gene Expr.* **12,** 245–257.
20. Jaiswal, N., Haynesworth, S.E., Caplan, A.I., and Bruder, S.P. (1997) Osteogenic differentiation of purified, culture-expanded human mesenchymal stem cells in vitro. *J. Cell. Biochem.* **64,** 295–312.
21. Fuchs, T.F., Petersen, W., Vordemvenne, T., Stange, R., Raschke, M., and Paletta, J.R. (2007) Influence of synovial fluid on human osteoblasts: an in vitro study. TheScientificWorldJOURNAL **7,** 2012–2020.
22. Frank, O., Heim, M., Jakob, M., Barbero, A., Schafer, D., Bendik, I., Dick, W., Heberer, M., and Martin, I. (2002) Real-time quantitative RT-PCR analysis of human bone marrow stromal cells during osteogenic differentiation in vitro. *J. Cell. Biochem.* **85,** 737–746.
23. Martin, I., Jakob, M., Schafer, D., Dick, W., Spagnoli, G., and Heberer, M. (2001) Quantitative analysis of gene expression in human articular cartilage from normal and osteoarthritic joints. *Osteoarthritis Cartilage* **9,** 112–118.
24. Waris, E., Ashammakhi, N., Kaarela, O., Raatikainen, T., and Vasenius, J. (2004) Use of bioabsorbable osteofixation devices in the hand. *J. Hand Surg. [Br.]* **29,** 590–598.
25. Suuronen, R., Kallela, I., and Lindqvist, C. (2000) Bioabsorbable plates and screws: current state of the art in facial fracture repair. *J. Craniomaxillofac. Trauma* **6,** 19–27; discussion 28–30.
26. Tanaka, N., Hirose, K., Sakahashi, H., Ishima, T., and Ishii, S. (2004) Usefulness of bioabsorbable thread pins after resection arthroplasty for rheumatoid forefoot reconstruction. *Foot Ankle Int.* **25,** 496–502.
27. Giavaresi, G., Tschon, M., Borsari, V., Daly, J.H., Liggat, J.J., Fini, M., Bonazzi, V., Nicolini, A., Carpi, A., Morra, M., Cassinelli, C., and Giardino, R. (2004) New polymers for drug delivery systems in orthopaedics: in vivo biocompatibility evaluation. *Biomed. Pharmacother.* **58,** 411–417.
28. Dersch, R., Liu, T., Schaper, A.K., Greiner, A., and Wendorff, J.H. (2003) Electrospun nanofibers: internal structure and intrinsic orientation. *J Polymer Sci. A Polymer Chem.* **41,** 545–553.
29. Zeng, J., Hou, H., Schaper, A., Wendorff, J.H., and Greiner, A. (2003) Poly-L-lactide nanofibers by electrospinning -- influence of solution viscosity and electrical conductivity on fiber diameter and fiber morphology. e-polymers, No 9.
30. Boudriot, U., Dersch, R., Greiner, A., and Wendorff, J.H. (2006) Electrospinning approaches toward scaffold engineering--a brief overview. *Artif. Organs* **30,** 785–792.
31. Boudriot, U., Goetz, B., Dersch, R., Greiner, A., and Wendorff, H.J. (2005) Role of Electrospun Nanofibers in Stem Cell Technologies and Tissue Engineering. Macromolecular Symposia 225. pp. 9–16.
32. Jahno, V.D., Ribeiro, G.B., dos Santos, L.A., Ligabue, R., Einloft, S., Ferreira, M.R., and Bombonato-Prado, K.F. (2007) Chemical synthesis and in vitro biocompatibility tests of poly (L-lactic acid). *J. Biomed. Mater. Res. A* **83,** 209–215.
33. Ishaug, S.L., Yaszemski, M.J., Bizios, R., and Mikos, A.G. (1994) Osteoblast function on synthetic biodegradable polymers. *J. Biomed. Mater. Res.* **28,** 1445–1453.
34. Hu, J., Liu, X., and Ma, P.X. (2008) Induction of osteoblast differentiation phenotype on poly(l-lactic acid) nanofibrous matrix. *Biomaterials* **29,** 3815–3821.
35. Ngiam, M., Liao, S., Patil, A.J., Cheng, Z., Yang, F., Gubler, M.J., Ramakrishna, S., and K., C.C. (2008) Fabrication of mineralized polymeric nanofibrous composites for bone graft materials. *Tissue Eng. Part A* [Epub ahead of print].
36. Venugopal, J.R., Low, S., Choon, A.T., Kumar, A.B., and Ramakrishna, S. (2008) Nanobioengineered electrospun composite nanofibers and osteoblasts for bone regeneration. *Artif. Organs* **32,** 388–397.
37. Shih, Y.R., Chen, C.N., Tsai, S.W., Wang, Y.J., and Lee, O.K. (2006) Growth of mesenchymal stem cells on electrospun type I collagen nanofibers. *Stem Cells* **24,** 2391–2397.
38. Li, W.J., Cooper, J.A., Jr., Mauck, R.L., and Tuan, R.S. (2006) Fabrication and characterization of six electrospun poly(alpha-hydroxy ester)-based fibrous scaffolds for tissue engineering applications. *Acta Biomater.* **2,** 377–385.
39. Maretschek, S., Greiner, A., and Kissel, T. (2008) Electrospun biodegradable nanofiber nonwovens for controlled release of proteins. *J. Control. Release* **127,** 180–187.
40. Kim, K., Luu, Y.K., Chang, C., Fang, D., Hsiao, B.S., Chu, B., and Hadjiargyrou, M. (2004) Incorporation and controlled release of a hydrophilic antibiotic using poly(lactide-co-glycolide)-based electrospun nanofibrous scaffolds. *J. Control. Release* **98,** 47–56.
41. Li, D., Frey, M.W., and Baeumner, A.J. (2006) Electrospun polylactic acid nanofiber membranes as substrates for biosensor assemblies. *J. Membr. Sci.* **279,** 354–363.
42. Nie, H., Soh, B.W., Fu, Y.C., and Wang, C.H. (2008) Three-dimensional fibrous PLGA/HAp composite scaffold for BMP-2 delivery. *Biotechnol. Bioeng.* **99,** 223–234.
43. Fu, Y.C., Nie, H., Ho, M.L., Wang, C.K., and Wang, C.H. (2008) Optimized bone regeneration based on sustained release from three-dimensional fibrous PLGA/HAp composite scaffolds loaded with BMP-2. *Biotechnol. Bioeng.* **99,** 996–1006.
44. Patel, S., Kurpinski, K., Quigley, R., Gao, H., Hsiao, B.S., Poo, M.M., and Li, S. (2007) Bioactive nanofibers: synergistic effects of nanotopography and chemical signaling on cell guidance. *Nano Lett.* **7,** 2122–2128.
45. Nur, E.K.A., Ahmed, I., Kamal, J., Babu, A.N., Schindler, M., and Meiners, S. (2008) Covalently attached FGF-2 to three-dimensional polyamide nanofibrillar surfaces demonstrates enhanced biological stability and activity. *Mol. Cell.*

*Biochem.* **309,** 157–166.

46. Hosseinkhani, H., Hosseinkhani, M., Khademhosseini, A., and Kobayashi, H. (2007) Bone regeneration through controlled release of bone morphogenetic protein-2 from 3-D tissue engineered nano-scaffold. *J. Control. Release* **117,** 380–386.
